# HPAStainR: a Bioconductor and Shiny app to query protein expression patterns in the Human Protein Atlas

**DOI:** 10.12688/f1000research.26771.2

**Published:** 2021-03-22

**Authors:** Tim O. Nieuwenhuis, Marc K. Halushka

**Affiliations:** 1 Department of Pathology, Johns Hopkins University School of Medicine Baltimore, Baltimore, MD, 21205, USA; 2 McKusick-Nathans Institute, Department of Genetic Medicine, Johns Hopkins University School of Medicine, Baltimore, MD, 21205, USA

**Keywords:** protein staining, Human Protein Atlas, marker genes, marker proteins, exploratory analysis

## Abstract

The Human Protein Atlas is a website of protein expression in human tissues. It is an excellent resource of tissue and cell type protein localization, but only allows the query of a single protein at a time. We introduce HPAStainR as a new Shiny app and Bioconductor/R package used to query the scored staining patterns in the Human Protein Atlas with multiple proteins/genes of interest. This allows the user to determine if an experimentally-generated protein/gene list associates with a particular cell type. We validated the tool using the Panglao Database cell type specific marker genes and a Genotype Expression (GTEx) tissue deconvolution dataset.  HPAStainR identified 92% of the Panglao cell types in the top quartile of confidence scores limited to tissue type of origin results. It also appropriately identified the correct cell types from the GTEx dataset. HPAStainR fills a gap in available bioinformatics tools to identify cell type protein expression patterns and can assist in establishing ground truths and exploratory analysis. HPAStainR is available from: https://32tim32.shinyapps.io/HPAStainR/

## Introduction

The Human Protein Atlas (HPA) has performed immunohistochemistry-based visual proteomics for over 15,313 proteins across 59 tissues. Within each tissue a number of different cell types have been scored for staining patterns by a group of pathologists. Therefore, there is a great amount of visual proteomic data that can be used to classify gene or protein lists into specific cell types ^1– 3^. Their website is designed to query one protein of interest at a time and there is no option to query a list of proteins to determine if that protein set is enriched in a particular cell type. This would be a useful feature to take advantage of this robust dataset. Other gene list tools such as Enrichr, which query multiple databases for associations, have not incorporated the HPA protein cell expression dataset into their tools ^4^. There are other R packages used to incorporate and query HPA data such as hpar ^5^, which allows for easy loading and querying of version controlled data and HPAnalyze ^6^ which has powerful visualization tools for protein levels. However, both packages lack the functionality of determining enriched proteins in the database.

We introduce HPAStainR ( https://32tim32.shinyapps.io/HPAStainR/), a Bioconductor R package and Shiny app developed to query the cell staining database of the HPA. HPAStainR allows a user to input a list of proteins/genes and returns a rank ordered list of cell types that are stained for the input list. HPAStainR is customizable, allowing the user the ability to include cancer or normal tissue data, change the HPA confidence levels, toggle the identification of what proteins from the list were detected, generate a p-value for how many cell type specific proteins are counted for a given cell type, and allow the downloading of the output as a comma separated (csv) file.

## Methods

### Implementation

The user interface of Shiny HPAStainR is made of a sidebar where one can input their protein/gene list, along with various options to customize the output of the Shiny app. The main panel consists of two tabs. The first tab is the output tab, where the DataTable from the user’s query is output. The second tab is informational giving the user a list of HPA cell types and how many proteins were tested/histologically scored in a given cell type.

The HPAStainR package is available on Bioconductor. The package shares all of the same functionality as the Shiny web application, including the ability to run the Shiny app locally and acquire all of the data to do so. This allows HPAStainR to be used as the Shiny app or incorporated into a local R pipeline.

### Operation

HPAStainR is an online Shiny app ^7^, available at http://shinyapps.io, and as a Bioconductor R Package ( https://bioconductor.org/) ^8^ with its source code available on GitHub ( https://github.com/tnieuwe/HPAStainR). The function has been tested on R version 3.6.1 and later. Minimal requirements are the same as RStudio’s system requirements [ https://bit.ly/2UqwXc6].

***Installation:*** The installation of the HPAStainR package can be done in R using the following commands:

> if (!requireNamespace("BiocManager", quietly = TRUE))

> install.packages("BiocManager")`

> BiocManager::install("HPAStainR")`

The remote-Shiny web application can be accessed via the following link: 

https://32tim32.shinyapps.io/HPAStainR/

Note: This analysis uses HPAStainR v.1.1.4 which is available on the HPAStainR Github using:

> install.packages("devtools")

> install_github("tnieuwe/HPAStainR")

And through the devel version of Bioconductor:

> if (!requireNamespace("BiocManager", quietly = TRUE))

> install.packages("BiocManager")

> BiocManager::install(version = 'devel ')

> BiocManager::install("HPAStainR")

The next Bioconductor version (3.13) is expected in April or May of 2021 and at that time HPAStainR v.1.2.0 will be released with all the changes in the devel. At that time the HPAStainR shiny app will be updated as well.

***Input:*** There are three required R objects for the main HPAStainR function to work and one optional data frame. The first two required objects are the public staining files from the HPA, which can be downloaded using the package and the `HPA_data_downloader()` function. If the argument `save_file` in `HPA_data_downloader()`is set to `TRUE` then the file will be dated and downloaded, and following runs will load the saved file. The third required input is either a vector of proteins or genes or a character list of proteins separated by a space, comma, or newline to be queried in HPAStainR. The optional data frame, used in the Shiny app version of HPAStainR, is a table that contains the percent of proteins that stained the tissue compared to the number of the proteins evaluated in the tissue, represented in Extended Table 1 ^9^, which can be generated using the `hpa_summary_maker()` function. This table demonstrates that not all cell types/tissues have the same number of proteins stained for.

***Output:*** If using `HPAStainR()`, a tibble containing the summarized detection of the input list of proteins or genes for each available cell type customized by the options selected before running the analysis. If using `shiny_HPAStainR()`, a shiny Datatable containing the data previously mentioned in the base `HPAStaiR()` output.

### HPA data distribution

**Figure 1.**
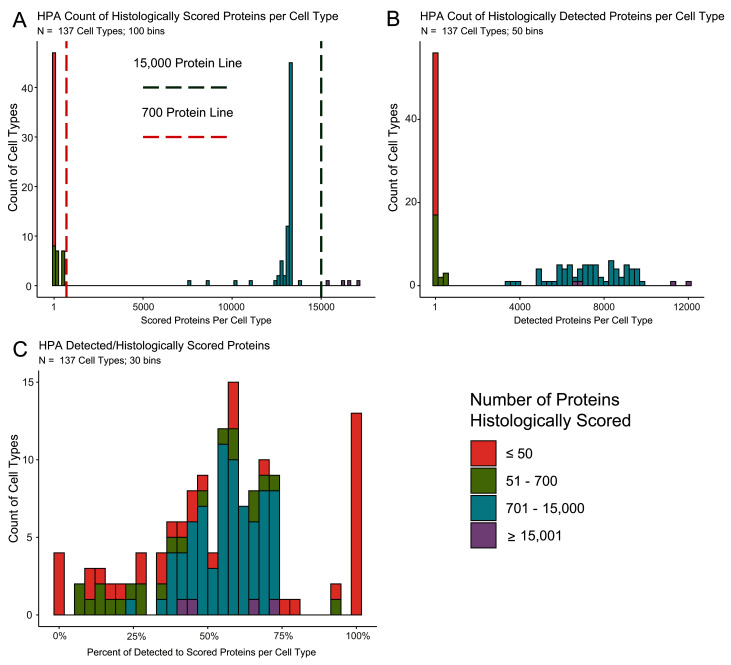
Histograms comparing how often proteins stain to how often they are evaluated in HPA. **A**. A histogram of the 137 cell types showing the amount of proteins histologically scored in each cell type. Four cell types were evaluated for >15,000 proteins (green line) and 61 for <700 proteins (red line). This bimodal distribution shows that there are nearly separate groups of cell types based on how often they are scored. **B**. A histogram of the 137 cell types on the amount of proteins that had positive staining in each cell type. This distribution reveals an expected skew, cell types that are more often histologically scored, tend to have a higher count of proteins detected. **C**. A histogram of the percent of positively stained proteins to the total amount of histologically scored proteins for the given cell type. The extreme ends of the distribution are populated by samples with less than 700 scored proteins revealing that seldom scored cell types staining frequencies are often artefacts.

Our analysis in this paper uses the 19.3 version of the Human Protein Atlas Data. Staining was scored by cell type in each tissue by a group of pathologists who rated the intensity in each evaluated cell type as “high, medium, low or not detected.” Not all cell types in all tissues were scored, nor were all cell types consistently evaluated. As a result, there are some caveats in the HPA data that should be noted. The distribution of how many proteins are histologically scored in each of the 137 cell types varies in HPA, such that not all results are equal. The number of proteins scored in cell types ranges from 1 in four substantia nigra cell types to over 17,000 in endometrial glandular cells ( Figure 1A; Extended Table 1 ^9^), impacting how often a protein is detected in a given cell type ( Figure 1B). The percent of stained to scored proteins demonstrates an enrichment at both extremes of the distribution ( Figure 1C). To highlight this discrepancy in testing, we have made the information in Extended Table 1 as an available tab on the Shiny app. This data can also be made using the `HPA_summary_maker()` function on normal tissue.

### Staining score calculation

The staining score calculation is an arbitrary measure of how well an input list of proteins are enriched for a particular cell type. A formal equation is below, but briefly, it is calculated based on the frequency and intensity of staining within a given cell type. Staining intensity is a percentage of high, medium, low, and not detected counts. The high percentage is multiplied by a value of 100, the medium percentage by 50, and the low percentage by 25, before adding all the results together to generate the final staining score. While arbitrary, we over-weighted high staining as the IHC was more robust and those proteins may better define the cell type. To illustrate the distribution of the staining score we generated 1,000 HPAStainR results on random genes, including the top 10 results from HPAStainR and all results, for random gene lists of sizes 10, 25, 50, and 100. As the number of genes increases the staining score distribution decreases. For the top 10 results from each run, ordered on staining score, the distribution appears to be normal (Extended Figure 1). Analysis of all staining data suggests a right skew (Extended Figure 2).

The model for the staining score equation is below where *t* is the total number of proteins from the list tested in the cell type, *h* is the number of proteins with high staining in the cell type, *m* is the number of proteins with medium staining in the cell type, and *l* is the number of proteins with low staining in the cell type.

$$\text{Staining}\;\text{Score}=\left(\frac{h\times 100}{t}\right)+\left(\frac{m\times 50}{t}\right)+\left(\frac{l\times 25}{t}\right)$$

### Confidence score calculation

The confidence score is unique to this paper, and only used for the comparison of the Panglao Database (PanglaoDB) cell types to HPA cell types. It is a modified version of the staining score adjusting for size of the protein list for each cell type from PanglaoDB. The confidence score calculation weights PanglaoDB cell types based on how many marker genes they have. Like the staining score, this score ranges from 0–100. The model for the equation is below, where *p* is the number of proteins tested, with a max *p* being 50 (standardizing the score range), and the staining score of the protein list in the cell type is represented by *s*.

$$\text{Confidence}\;\text{Score}=\frac{p\times s}{50}$$

### Cell type enriched p-value

While we utilized all expressed proteins in our staining score, we recognize that some proteins demonstrate cell type enrichment. For this analysis we generated the “enriched-protein p-value” based on either a Fisher’s Exact Test or χ ^2^ analysis. In this paper we used the results of the Fisher’s Exact Test.

**Figure 2.**
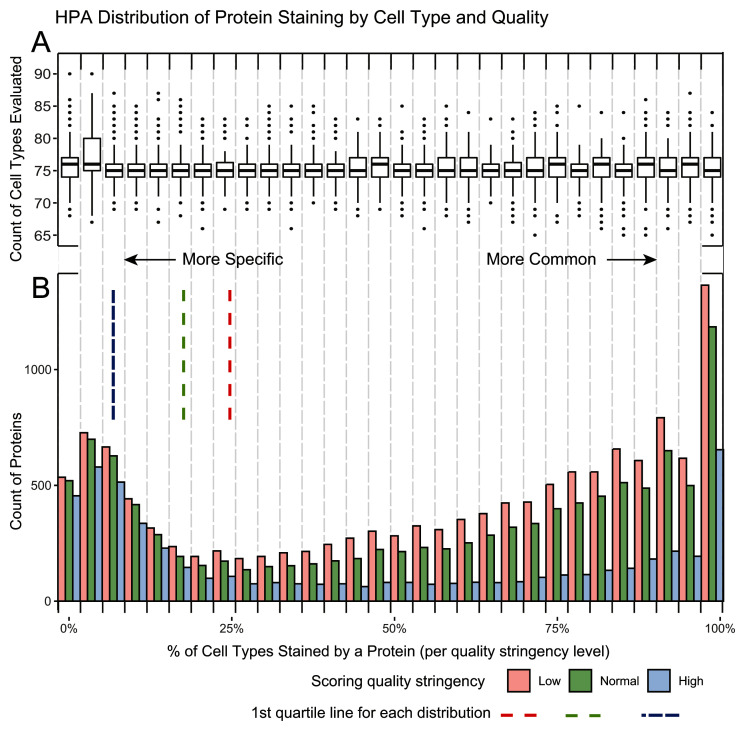
HPA distribution of protein staining by cell type and quality. **A**. A boxplot of the number of cell types evaluated per protein in each of the 30 histogram bins. The overall median of cell types is 75 proteins with the 1 ^st^ and 3 ^rd^ quartile being 74 and 77 proteins respectively. This illustrates that tissue enrichment of a protein is not an artifact of how often a protein is scored, as there is a similar level of testing across bins. **B**. A histogram demonstrating the percent of positive staining cells per protein. Three quality stringencies are given. The 1 ^st^ quartile lines demonstrate the specificity cut off of the distribution used at each stringency level. As expected, the number of proteins in the 1 ^st^ quartile increase with lower stringency. More commonly detected genes are more greatly affected by stringency when compared to rarely detected genes. There also appear to be a larger number of specific proteins (10%) than there are of semi-specific proteins (25%) in HPA, as the distribution from common to specific decreases before peaking again.

**Figure 3.**
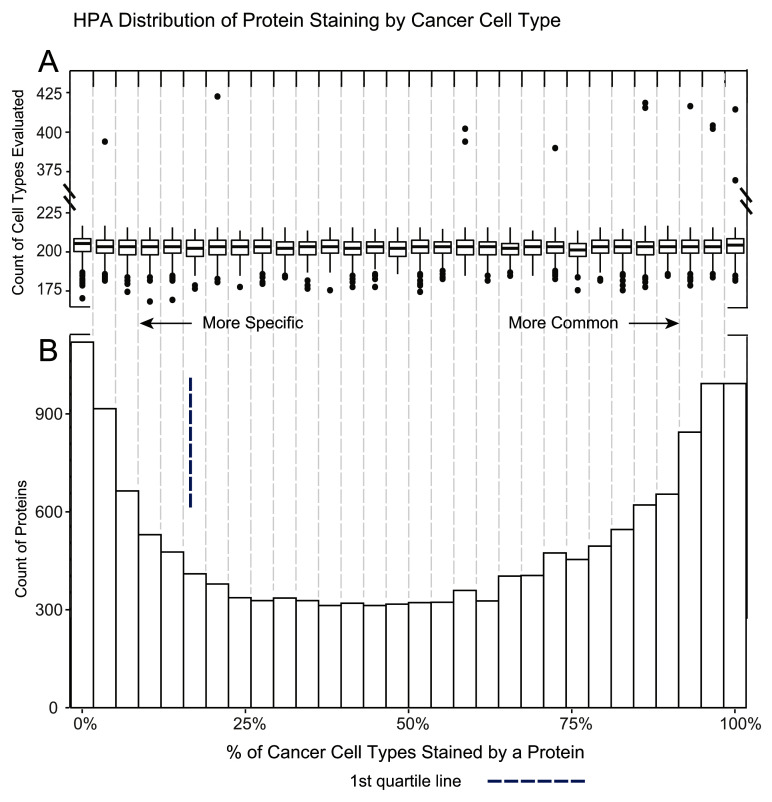
HPA distribution of protein staining by cancer cell type. **A**. A boxplot of the number of cell types evaluated per protein in each of the 30 histogram bins. The overall median of cancer cell types is 203 with the 1 ^st^ and 3 ^rd^ quartile of 199 and 207 respectively. A number of outliers with ~2x more cell types evaluated are noted. Besides frequently tested cancers, the distribution reveals proteins are evenly scored across cancers, regardless of how frequently they are tested. **B**. A histogram demonstrating the percent of positive staining cancer cells per protein. This distribution is different from normal tissue as some cancer samples of the same cancer type can positively stain for a protein while other samples will not. Similar to normal tissue there seems to be an increase of proteins in the extreme ends of the specificity distribution.

To calculate the enriched-protein p-value we generated a list of cell type enriched proteins for each level of stringency, low, normal and high. The stringency parameter filters the `Reliability` column from the normal tissue HPA dataset. This `Reliability` varies from “Enhanced,” “Supported,” “Approved,” to “Uncertain” in decreasing order of certainty (full descriptions of these labels are found here https://www.proteinatlas.org/about/assays+annotation). Low stringency includes all data, normal stringency includes “Enhanced,” “Supported,” and “Approved,” while high stringency only includes “Enhanced” and “Supported”. The cell type enriched protein list was generated by calculating a percentage of positively stained to evaluated proteins across each cell type to adjust for protein scoring frequency. This percentage generated our ‘enriched proteins’ list from the top quartile of enriched proteins (the proteins present in <25% of the evaluated cell types. The number of proteins were 3,275, 2,543, and 1,235 for low, normal, and high stringency respectively and 3,818 in cancer) (Extended Tables 2 and 3 ^9^; Figure 2 and Figure 3). The Fisher’s Exact Test analysis was based on the staining presence/absence of ‘enriched proteins’ for a given HPA cell vs presence/absence of proteins from a protein list query. For all experiments in this paper, stringency was set to normal. 

All code for the package and the analysis can be found on GitHub at https://github.com/tnieuwe/HPAStainR and https://github.com/tnieuwe/HPAstainR_dev_paper, respectively.

## Use cases

### HPA functionality

**Figure 4.**
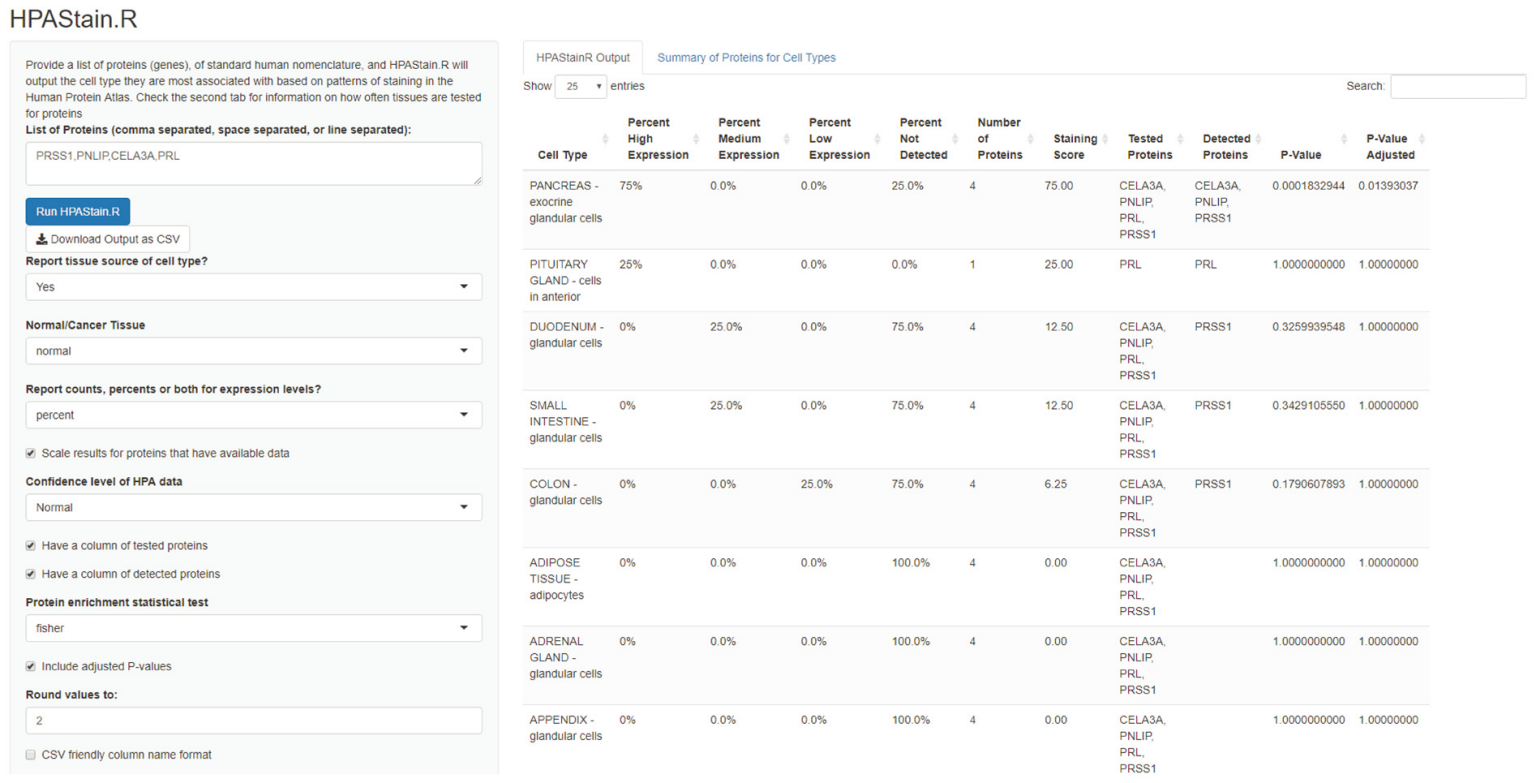
A screen shot of the user interface of HPAStainR. A list of comma, space, or line separated proteins or genes are inputted on the left column. Multiple customizations are available for users below to optimize the search parameters for their query of interest. Data is outputted to the right, and further information about the cell types and how many proteins were histologically scored per cell type are available as a second tab.

HPAStainR uses the publicly available HPA cell type histologically scored staining data to identify the top cell type matches to a queried protein/gene list. It ranks cell types on a 0 to 100 “staining score” ( Figure 4). This score is based on the pathologist annotated staining intensity (high, medium, low) of each protein/gene in the query list for each HPA cell type, as a percent of the total number of proteins/genes queried (see Methods). For example, a query of the pancreatic enzymes PRSS1, PNLIP, and CELA3A, along with the protein PRL, would identify “pancreas exocrine glandular cells” as the top hit with a staining score of 75 due to the high staining intensity in three proteins and negative staining of the fourth protein. The second hit would be the “pituitary gland cells in anterior” due to PRL’s high expression in that cell type (score of 25), followed by “intestinal glandular cells” which only have medium staining of PRSS1 (score of 12.5).

### The Panglao Database

**Figure 5.**
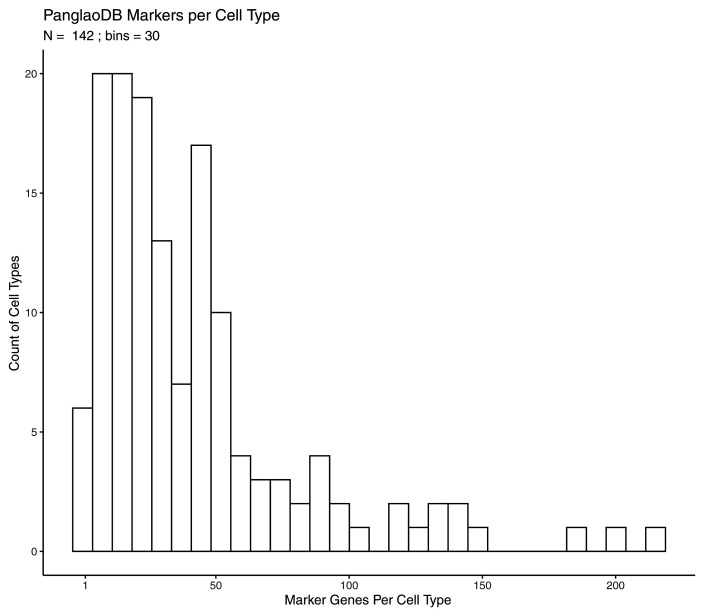
PanglaoDB markers per cell type. Histogram of the number of marker genes used to define 142 different Panglao cell types.

To show the functionality of the Shiny app we applied HPAStainR to the Panglao Database, a hub of community-curated cell type markers from single cell data ^10^. We wanted to investigate how well HPAStainR would mark the cell types based on PanglaoDB’s annotations. We downloaded a tsv file of PanglaoDB’s cell type gene marker data, and parsed it down to only human protein coding marker genes. We assayed 146 human cell types and their 3,661 marker genes through HPAStainR. The number of marker genes per cell type in PanglaoDB are variable, ranging from one marker in trophoblast stem cells to 216 in interneurons. A histogram of markers per cell type showing the distribution can be found in Figure 5.

### HPAStainR identified many of the cell types in PanglaoDB

To perform analyses between multiple runs of HPAStainR and PanglaoDB, we generated a “confidence score,” a value (theoretical 0–100) that corrected for the staining score’s determination using an additional feature of how many proteins were evaluated (see Methods). This score weighted cell types with multiple marker proteins staining over cell types with a single or fewer marker proteins. Thus, the confidence score allowed us to rank the cell types based on both staining and depth of data.

HPAStainR is agnostic to the source of a protein/gene list. Therefore, an identification of equivalent cell types across two methods provides strong evidence of HPAStainR’s usefulness. Specific protein lists, corresponding to the 146 cell types were evaluated from PanglaoDB in HPAStainR with the top HPA cell types identified for each. To cover both potential user needs, we included in our PanglaoDB output both the top result of HPAStainR and the top result in the appropriate tissue. The confidence score across these comparisons, generated on HPAStainR data, ranged from 1.5 to 66.75. The results of the 146 cell types were divided into quartiles (Qs) based on the confidence score. The average number of proteins associated with a PanglaoDB cell type used to identify the top HPA cell type strongly correlated with the quartile (76.3; 55.7; 23.9; 7.7 proteins in Q1 to Q4, respectively). In the top quartile of scores, 75% (27/36) of cells matched between PanglaoDB and HPA. Of the nine that were not a perfect match, six matched the top hit when limited by tissue type. Of the remaining Q1 PanglaoDB cell types; liver kupffer cells (a type of macrophage), mesothelial cells, and embryonic stem cells, none had matching cell types in the HPA ^11^. 

A subset of this analysis can be seen below in Table 1 with the full results being in Extended Table 4 ^9^. Results were ranked by confidence score, with a strong correlation of higher confidence scores to more accurate cell type assignments between PanglaoDB and HPA. An interesting example are chondrocytes, where the top stained score (27.25) was to TONSIL squamous epithelial cells and the top tissue specific cell type was SOFT TISSUE - chondrocytes (20.75). In addition to the stain score, HPAStainR provides a p-value (and Holm adjusted p-value) based on a separate metric based on cell type specific/enriched protein expression (see Methods). Although tonsil squamous epithelial cells is the top HPAStainR result, the adjusted enriched protein p-value was 1.0 (nonsignificant) while it was p=2.5E-04 for the chondrocytes, indicating cell-enriched proteins favored the correct match.

**Table 1.** A subset of 10 HPAStainR results of PanglaoDB cell type marker queries. Both the overall top HPAStainR result and a tissue-specific result is given. The “Select Tissues” results are from a search performed for the PanglaoDB cell type only within the matched tissue type (ALL CAPITALIZED) in HPA.

PanglaoDB Cell Type	Confidence Score	Top HPAStainR Result	Top Result Stained Score	Top Adjusted P-value	Select Tissue Top Result	Select Tissue Top Stained Score	Select Tissue Adjusted P-value
KIDNEY proximal tubule cells	66.75	KIDNEY - cells in tubules	66.75	1.59E-18	KIDNEY - cells in tubules	66.75	1.59E-18
HEART MUSCLE cardiomyocytes	61	HEART MUSCLE - myocytes	61	1.29E-33	HEART MUSCLE - myocytes	61	1.29E-33
IMMUNE SYSTEM neutrophils	60	BONE MARROW - hematopoietic cells	60	9.99E-13	BONE MARROW - hematopoietic cells	60	9.99E-13
OLFACTORY SYSTEM olfactory epithelial cells	51.25	BRONCHUS - respiratory epithelial cells	51.25	2.17E-05	NASOPHARYNX - respiratory epithelial cells	39.75	0.00076
CONNECTIVE TISSUE adipocytes	33.75	KIDNEY - cells in tubules	33.75	1	ADIPOSE TISSUE - adipocytes	24.25	0.02353
CONNECTIVE TISSUE chondrocytes	27.25	TONSIL - squamous epithelial cells	27.25	1	SOFT TISSUE - chondrocytes	20.75	0.00025
REPRODUCTIVE granulosa cells	13.6	PLACENTA - trophoblastic cells	42.5	0.00174	OVARY - follicle cells	25	1
HEART MUSCLE purkinje fiber cells	5.775	CAUDATE - neuronal cells	57.75	1	HEART MUSCLE - myocytes	0	1
BRAIN cholinergic neurons	5.25	DUODENUM - glandular cells	37.5	1	CEREBRAL CORTEX - neuronal cells	31.25	1
EMBRYO trophoblast progenitor cells	3	PLACENTA - trophoblastic cells	50	1	tissue not found	NA	NA

### HPAStainR can help determine cell type populations in bulk RNA sequencing

**Figure 6.**
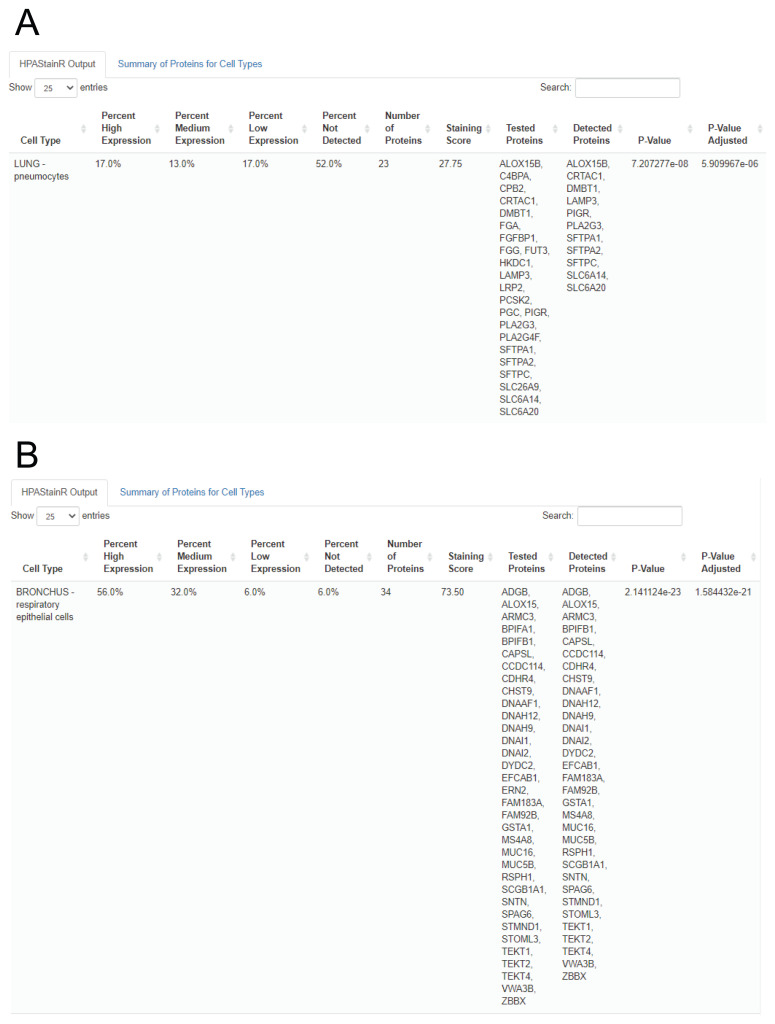
Top HPAStainR results from high variance lung clusters. **A**. Represents the output when a list of genes associated with pneumocytes are used as input. **B**. Represents the output when a list of genes associated with the bronchial epithelium are used as input.

We then demonstrated the functionality of HPAStainR in bulk datasets. We utilized the variable gene expression data from the Genotype Expression (GTEx) dataset that we had previously uncovered as being driven by variation in pneumocytes or the presence of bronchial epithelium ^12^. There were 33 genes identified in the pneumocyte cluster and 70 genes in the bronchial epithelium cluster. HPAStainR was applied separately to both lists and found the top results to be lung pneumocytes and bronchus respiratory epithelial cells respectively ( Figure 6A and 6B; Extended Tables 5 and 6 ^9^). Therefore, across both single cell and bulk gene expression data, we have identified useful functionality to HPAStainR.

## Conclusion

HPAStainR fills a small gap in our knowledge base by allowing for the query of gene/protein lists against the cellular protein expression pattern data of HPA. As datasets of single cell RNA sequencing analysis become available, it is useful to have a tool to correlate these individual cellular transcriptomic gene profiles with translated protein expression patterns. We have also shown the tool can recapitulate bulk RNA sequencing findings making it a valuable tool to understand the cellular composition of a sample. The HPA is an excellent resource to observe staining patterns within cells across tissues for proteins of interest. The limitations of the study are the quality of the staining across all HPA tissues and the quality/consistency of the pathology scoring of the tissues ^13^. Both of these may impact the scoring achieved for any given query. HPAStainR is a new valuable resource to accelerate exploratory and ground truth queries in the HPA cell type protein staining data.

## Data availability

### Underlying data

The data from PanglaoDB was downloaded at https://panglaodb.se/markers/PanglaoDB_markers_27_Mar_2020.tsv.gz (last updated March 27th 2020).

Human Protein Atlas normal tissue and cancer tissue data was acquired from the website: https://www.proteinatlas.org/about/download (last visited March 28 ^th^ 2020)

### Extended data

Harvard Dataverse: HPAStainR – A Bioconductor and shiny app to query protein expression patterns in the Human Protein Atlas, https://doi.org/10.7910/DVN/CL5ZTA ^9^.

This project contains the following extended data:

- **Extended Figure 1. The distribution of the top 10 results from 1,000 permutations of HPAStainR.** **A**. A histogram of the top 10 results selecting 10 genes in each permutation. **B**. A histogram of the top 10 results selecting 25 genes in each permutation. **A**. A histogram of the top 10 results selecting 50 genes in each permutation. **A**. A histogram of the top 10 results selecting 100 genes in each permutation.
- **Extended Figure 2. The distribution of all results from 1,000 permutations of HPAStainR.** **A**. A histogram of all results selecting 10 genes in each permutation. **B**. A histogram of all results selecting 25 genes in each permutation. **A**. A histogram of all results selecting 50 genes in each permutation. **A**. A histogram of all results selecting 100 genes in each permutation.
- **Extended Table 1**. **The number of proteins evaluated and positively stained for each cell type.** For each tissue cell combination the number of proteins being positively scored over the number of times proteins are evaluated. These are categorically grouped on the amount of proteins evaluated.
- **Extended Table 2. The rarity for proteins in normal tissue across different filters.** For each gene in normal tissue that was detected, the percent of how often proteins stained compared to how often they were histologically scored based on three quality filters, low, normal and high. Each protein is also labeled if it is considered rare or not in a given tissue based on if its percentage was in the bottom 1 ^st^ quartile of the distribution for each quality filter. NA indicates that the protein never reached the threshold to be counted in a given filter level. Proteins that never positively stained were not included.
- **Extended Table 3. The rarity of proteins in cancer samples.** The percent positive staining of 15,301 in cancer cells. The quartile of proteins with the lowest values were indicated as rare. Note, there is no quality filter for cancer thus different cancer samples from the same type of cancer can have different staining patterns.
- **Extended Table 4. The extended HPAStainR output from Table 1.**
- **Extended Table 5. HPAStainR output of cluster A from McCall *et al*.** The full results of HPAStainR when running cluster A of McCall *et al*. through the package. Pneumocytes were expected and observed.
- **Extended Table 6. HPAStainR output of cluster B from McCall *et al*.** The full results of HPAStainR when running cluster B of McCall *et al*. through the package. Bronchial epithelial cells were expected and observed.

Data are available under the terms of the Creative Commons Zero "No rights reserved" data waiver (CC0 1.0 Public domain dedication).

## Software availability

Software available from: https://32tim32.shinyapps.io/HPAStainR/

Bioconductor package available from: https://doi.org/doi:10.18129/B9.bioc.HPAStainR

Source code available from: https://github.com/tnieuwe/HPAStainR

Archived source code as at time of publication: https://doi.org/10.5281/zenodo.4594755 ^14^.

Software license: Artistic-2.0

Analysis code available from: https://github.com/tnieuwe/HPAstainR_dev_paper

Archived analysis code as at time of publication: https://doi.org/10.5281/zenodo.4594672 ^15^.

License: Artistic-2.0
