# Supplementary material for: HPAStainR: a Bioconductor and Shiny app to query protein expression patterns in the Human Protein Atlas
Source: F1000Res. 2021 Mar 22;9:1210. Originally published 2020 Oct 8. [Version 2] doi: 10.12688/f1000research.26771.2 (PMC7802108; doi:10.12688/f1000research.26771.2)
Supplement: Peer review history

# Peer review: Reviewer response for version 2

**DOI:** 10.5256/f1000research.55429.r81836

**Published:** 2021-04-07

**Authors:** Yasin Kaymaz

**Affiliations:** 1 Harvard University, Boston, MA, USA

I would like to thank all authors for addressing my concerns. I have no further comment about the revised version.

Are the conclusions about the tool and its performance adequately supported by the findings presented in the article?

Yes

Is the rationale for developing the new software tool clearly explained?

Yes

Is the description of the software tool technically sound?

Partly

Are sufficient details of the code, methods and analysis (if applicable) provided to allow replication of the software development and its use by others?

Yes

Is sufficient information provided to allow interpretation of the expected output datasets and any results generated using the tool?

Yes

Reviewer Expertise:

Bioinformatics particularly in RNA-seq, single-cell genomics, and algorithm dev. areas.

I confirm that I have read this submission and believe that I have an appropriate level of expertise to confirm that it is of an acceptable scientific standard.

# Peer review: Reviewer response for version 2

**DOI:** 10.5256/f1000research.55429.r81837

**Published:** 2021-04-06

**Authors:** Laurent Gatto

**Affiliations:** 1 De Duve Institute, UCLouvain, Brussels, Belgium

I have no further comments to make.

Are the conclusions about the tool and its performance adequately supported by the findings presented in the article?

Yes

Is the rationale for developing the new software tool clearly explained?

Yes

Is the description of the software tool technically sound?

Partly

Are sufficient details of the code, methods and analysis (if applicable) provided to allow replication of the software development and its use by others?

Yes

Is sufficient information provided to allow interpretation of the expected output datasets and any results generated using the tool?

Partly

Reviewer Expertise:

Computational biology, proteomics, genomics, research software development, open and reproducible research.

I confirm that I have read this submission and believe that I have an appropriate level of expertise to confirm that it is of an acceptable scientific standard.

# Peer review: Reviewer response for version 2

**DOI:** 10.5256/f1000research.55429.r81838

**Published:** 2021-03-22

**Authors:** Mazdak Salavati

**Affiliations:** 1 The Roslin Institute and Royal (Dick) School of Veterinary Studies, University of Edinburgh, Edinburgh, UK

I have no further comments.

Are the conclusions about the tool and its performance adequately supported by the findings presented in the article?

Yes

Is the rationale for developing the new software tool clearly explained?

Yes

Is the description of the software tool technically sound?

Yes

Are sufficient details of the code, methods and analysis (if applicable) provided to allow replication of the software development and its use by others?

Yes

Is sufficient information provided to allow interpretation of the expected output datasets and any results generated using the tool?

Yes

Reviewer Expertise:

Genetics and Genomics, Bioinfomatics, Cell biology.

I confirm that I have read this submission and believe that I have an appropriate level of expertise to confirm that it is of an acceptable scientific standard.

# Peer review: Reviewer response for version 1

**DOI:** 10.5256/f1000research.29559.r72699

**Published:** 2021-01-11

**Authors:** Yasin Kaymaz

**Affiliations:** 1 Harvard University, Boston, MA, USA

Authors introduce a new R package called HPAStainR for quickly identifying putative cell types given a list of protein or gene names by searching these through the Human Protein Atlas database. HPAStainR has been developed to allow users to query multiple gene/protein names in the HPA database and return an ordered list of cell types for which the query list might be enriched. They also make use of the Panglao single-cell expression database to validate their predictions. The main purpose of the tool addresses a need in the field and should be encouraged. However, I would like to mention some of my concerns below about the basis of the tool and the structure of the manuscript. 

 It has been mentioned that there were two main input files from the HPA database which can be downloaded with the ‘HPA_data_downloader’ function. Do you need to download all the data from the HPA for each analysis? Is this really necessary? Can't you do this on the fly? 

 The staining score seems to be constructed a bit arbitrarily. Based on staining intensity (high, medium, and low), scores are weighted with some arbitrary constant values and normalized by the total query number. But I wonder if there is any skew in this scoring scheme in case extreme queries are tried, such as all high with many proteins or all low, etc. Especially, given the highly non-uniform scoring distribution in the HPA database (Figure 1).

 Confidence score? Why not combine this equation with the staining score? The point of keeping them as separate metrics seems a bit vague. Also, a bit confusing from the user standpoint. 

 Do the authors use the cell type enrichment p-value in the enrichment at all? If so, it is not obvious from the text.

 Figure 1, 2, and 3;

 The actual purpose of these figures should be clearly explained in the main text. It is hard to get it unless staring at them for a while. Also, these first three figures are not directly related to the tool explained here. They are rather some statistics showing the key points of HPA data. I would recommend replacing these with more directly related graphs demonstrating the performance of the HPAStainR and including the current ones as supplemental data.

Are the conclusions about the tool and its performance adequately supported by the findings presented in the article?

Yes

Is the rationale for developing the new software tool clearly explained?

Yes

Is the description of the software tool technically sound?

Partly

Are sufficient details of the code, methods and analysis (if applicable) provided to allow replication of the software development and its use by others?

Yes

Is sufficient information provided to allow interpretation of the expected output datasets and any results generated using the tool?

Yes

Reviewer Expertise:

Bioinformatics particularly in RNA-seq, single-cell genomics, and algorithm dev. areas.

I confirm that I have read this submission and believe that I have an appropriate level of expertise to confirm that it is of an acceptable scientific standard, however I have significant reservations, as outlined above.

# Response

**Published:** 2021-03-12

**Authors:** Tim Nieuwenhuis

**Affiliations:** Johns Hopkins School of Medicine, USA

Thank you for taking the time to review this paper and the associated package, below is a response to your concerns and the changes we’ve made because of them.

**1. … Do you need to download all the data from the HPA for each analysis? Is this really necessary? Can't you do this on the fly?**

 In the current version of `HPA_data_downloader()`, as long as the parameter `save_file` is set to `TRUE`, it will only require you to download the files once. The next release of the package (v1.1.4 available on the main branch at https://github.com/tnieuwe/HPAStainR) includes an updated version of the function that marks the download date of the files, and also allows the user to select which downloaded file they want to use in their analysis for backward reproducibility.

**2. The staining score seems to be constructed a bit arbitrarily…**

 We acknowledge the somewhat arbitrary nature of the equation, although it is based on prior histology scoring methods more common in histopathology studies. Additionally, we have generated a distribution of 1,000 HPAStainR results on randomly selected genes, including the top 10 results from HPAStainR and all results, for random gene lists of sizes 10, 25, 50, and 100, these are found as Extended Figures 1 and 2. Our findings show the increased number of genes results in lower staining scores. Regardless, for the top 10 results, the distribution appears to be normal. Analysis of all staining data suggests a right skew. We were unable to create an extreme skew, but we cannot entirely exclude it for some unique queries.

**3. Why not combine the confidence score with the staining score?**

 The reason that they are separate is because the confidence score is simply a scaled staining score that is only used for PanglaoDB-HPAStainR analyses. The context we used the confidence score was strictly for testing how well proteins considered marker genes in PanglaoDB mark the equivalent cell types in HPA. This was described in greater detail to reviewer 2’s query.  

**4. Do the authors use the cell type enrichment pvalue at all?**

 Yes, in the original manuscript we use the p-value at the end of the **HPAStainR identified many of the cell types in PanglaoDB **section. We also show it in figure 6, and in the updated version of the manuscript, we now include it in table 1. Also, the p-values have changed as the analysis has been updated from a X ^2^ analysis to a Fisher’s Exact Test.

**5. The purpose of these figures should be clearly explained in the main text.**

 We have added further information in the figure legends to better clarify what the figures represent. HPAStainR does not have an output that lends itself to a graphical representation, however, we have added two extended figures to the manuscript showing the distribution of the staining score in various random samplings as noted above.

# Peer review: Reviewer response for version 1

**DOI:** 10.5256/f1000research.29559.r72698

**Published:** 2020-11-11

**Authors:** Laurent Gatto

**Affiliations:** 1 De Duve Institute, UCLouvain, Brussels, Belgium

Nieuwenhuis and Halushka describe the HPAStainR package, recently released as part of the Bioconductor project. HPAStainR uses protein immunostraining data from the Human Protein Atlas to asses whether a user-provided list of proteins or genes is associated with a particular cell type.

 ## Introduction

 The authors fail to cite other Biocondcutor packages related to the Human Protein Atlas, namely hpar (in Bioconductor for 8 years) and HPAnalyze (in Bioconductor for 2 years). While the functionalities of these packages are different (but see below), citing similar packages in Bioconductor seems very relevant for an paper in the Bioconductor gateway.

 (Note that I am the author of the hpar package)

 ## Methods: Operation

- Installation instructions should not refer to the development version of the package, but instruct users to install and use the release version. For two reasons: first the development of a package doesn't guarantee any stability, second it puts additional burden on the user to (1) potential have to install the development version of R and (2) end up with installing _all_ Bioconductor development packages. Given that package is released now, the installation instructions should absolutely be updated accordingly.
-  Please fix code formatting.

Input:

-  The `HPA_data_downloader()` function is used to download 2 datasets. Note that the 'hpar' package could have been a Bioconductor package to integrate with here, so as to avoid repeated downloading of the data and/or to provide some reproducibility in the analyses (see below for details on this).

```

 > ## Executed on the 10 November at 20:01

 > HPA_data <- HPA_data_downloader(tissue_type = 'both', save_file = FALSE)

 > HPA_out <- HPAStainR(c('PRSS1', 'PNLIP', 'CELA3A', 'PRL'),

                        HPA_data$hpa_dat,

                        HPA_data$cancer_dat,

                        'both')

 ```

 ```

 > library(hpar)

 > packageVersion("hpar") ## ‘1.32.1’

 > data(hpaCancer) ## load the data

 > data(hpaNormalTissue)

 > all.equal(hpaNormalTissue, HPA_data$hpa_dat)

 [1] TRUE

 > all.equal(hpaCancer, HPA_data$cancer_dat, check.attributes = FALSE) ## different colnames only

 [1] TRUE

 > ## BUT see below

 > HPA_out2 <- HPAStainR(c('PRSS1', 'PNLIP', 'CELA3A', 'PRL'),

                         hpaNormalTissue,

                         hpaCancer,

                         'both')

 ```

 There are generally two approaches when it comes to using data from remote resources:

1. Download the data on the fly, which allows to use the very latest version of the data, but at the expense of lack of reproducibility/tracking. Indeed, the results can unexpectedly change from one day to another. This would be to option in the HPAStainR package, as well as other Bioconductor packages such as rols (that queries various ontologies, including GO).
2. Packaging and versioning data to guarantee tracking and reproducibility of the analysis. This is for instance the option provided by hpar (latest hpar release, version 1.32.1, provides HPA data version 19.3, dated 2020/03/06). Other Bioconductor package that offer this solution are GO.db (package GO), and many other Bioconductor data packages.

It would be useful for HPAStainR to make these assumptions explicit and to document how to track results: using hpar or manually (and documenting!) storing the tables downloaded using `HPA_data_downloader()`.

 Returning to the reproducibility of the results, despite identical input data (except for the cancer data column names), it is intriguing that the results aren't identical. There are three cell types that have different p-values/adjusted p-values.

 ```

 > all.equal(HPA_out, HPA_out2)

 [1] "Component “p_val”: 2 string mismatches"  

 [2] "Component “p_val_adj”: 1 string mismatch"

 > which(HPA_out$p_val != HPA_out2$p_val)

 [1] 4 5

 > which(HPA_out$p_val_adj != HPA_out2$p_val_adj)

 [1] 1

 > HPA_out[c(1, 4, 5), c("cell_type", "p_val", "p_val_adj")]

 # A tibble: 3 x 3

   cell_type                           p_val  p_val_adj

                                        

 1 PANCREAS - exocrine glandular cells <0.005 0.076    

 2 SMALL INTESTINE - glandular cells   0.37   1.000    

 3 COLON - glandular cells             0.17   1.000

 > HPA_out2[c(1, 4, 5), c("cell_type", "p_val", "p_val_adj")]

 # A tibble: 3 x 3

   cell_type                           p_val  p_val_adj

                                        

 1 PANCREAS - exocrine glandular cells <0.005 0.038    

 2 SMALL INTESTINE - glandular cells   0.34   1.000    

 3 COLON - glandular cells             0.18   1.000    

 ```

 It would be interesting for the authors to investigate this, given that the PANCREAS - exocrine glandular cells change from non-significant to significant.

- 'hpa_summary_maker.R` must be `HPA_summary_maker()'.

Output:

- Running 'HPAStainR()' as indicated in the man page returns a tibble. It is unclear what the "Shiny DataTable" output in the text refers to. I couldn't find any further information in the man page. Did the authors possibly mean the 'shiny_HPAStainR()' function? Anyway, the two function should be mentioned in the manuscript.
- Running that very same example, the output table is incoherent in the mode of the variables: p-values (p_val) and adjusted p-values (p_val_adj) are encoded as characters.

## HPA data distribution

 The data discussed in 'HPA data distribution' and available in Extended Table 1 seems to be the same one as returned by the 'HPA_summary_maker()' function. Please mention this explicitly, to allow users to easily generate this table for different data.

 ## Confidence score calculation

 It isn't clear why the PanglaoDB needs an additional confidence score, or why it wouldn't be relevant or useful in other contexts.

 ## Cell type enriched p-value

 It isn't clear what is refereed to by low, normal and medium stringency. Based on the straining score calculation equation, it seems to be related to the low, high, medium staining intensity. Please define the notion of stringency.

 ## The Panglao Database

 In Table 1 and in the 'HPAStainR identified many of the cell types in PanglaoDB' section, the authors make use of the confidence score instead of the p-values to support their validation. Why don't they make use of that p-value, advertised in the previous 'Cell type enriched p-value' section?

 ##  HPAStainR can help determine cell type populations in bulk RNA sequencing

 The authors show the first hits, matching the expected cell types. Are there any other cell types that match with an adjusted p-value < 0.05?

 ## Software availability

 The authors mention that the analysis code is available from: https://github.com/tnieuwe/HPAstainR_dev_paper. This repositories however contains a lot of 'old' files ('old_vignette', 'old_files', 'old_versions'), including what appears an old version of the package in 'package_HPAstainR'. Version control is the ideal tool to store and track files over time, and dedicated version can be specifically tagged or released.

 Are these old version relevant? What are the differences with the more recent analyses?

Are the conclusions about the tool and its performance adequately supported by the findings presented in the article?

Yes

Is the rationale for developing the new software tool clearly explained?

Yes

Is the description of the software tool technically sound?

Partly

Are sufficient details of the code, methods and analysis (if applicable) provided to allow replication of the software development and its use by others?

Yes

Is sufficient information provided to allow interpretation of the expected output datasets and any results generated using the tool?

Partly

Reviewer Expertise:

Computational biology, proteomics, genomics, research software development, open and reproducible research.

I confirm that I have read this submission and believe that I have an appropriate level of expertise to confirm that it is of an acceptable scientific standard, however I have significant reservations, as outlined above.

# Response

**Published:** 2021-03-12

**Authors:** Tim Nieuwenhuis

**Affiliations:** Johns Hopkins School of Medicine, USA

We thank you for your time reviewing our package. Below we have individual responses to your queries formatted the same way as your review.

 ## Introduction

 Thank you for pointing out our oversight. We have included, in the newest version of the paper, a citation for both hpar and HPAnalyze. We will discuss the incorporation of hpar into our package later in this response.

 ## Methods

 Operation:

 In the new version, we have updated this section to properly reflect the release of HPAStainR and the code, properly formatted, to download said library.

 Input:

 HPAStainR’s next release, currently available on the main branch of https://github.com/tnieuwe/HPAStainR and the devel version of Bioconductor, will include a vignette section on how to use hpar’s data in HPAStainR. We are currently keeping the function `HPA_data_downloader()` as its one benefit over hpar is that it gives access to the most recent data if the semi-annual Bioconductor release doesn’t pair with the HPA release.

 In response to the remote data comment, we have also updated the package (v1.1.4) on the master branch for future release (https://github.com/tnieuwe/HPAStainR). The changes to `HPA_data_downloader()` includes the following:

  

1. The name of the file and function has changed from `hpa_data_downloader()` to `HPA_data_downloader()`
2. Every time a user downloads and saves the HPA files, the date of the download is provided.
3. To help in version control there are three new parameters that assist in maintaining reproducibility:
  1. `version_date_normal`: This parameter allows the user to insert a date string in YYYY-MM-DD format to select a normal tissue file downloaded on the respective date. If a date is not supplied the default argument is “last” which will find the latest version of the file.
  2. `version_date_cancer`: The same as version date normal, but for the pathology/cancer file.
  3. `force_download`: An argument forcing the re-downloading of files from the HPA website. The purpose of this argument is to allow the user to update their local files, as `HPA_data_downloader()` by default will use local files over downloading said files again.

We investigated the incongruency between your run of HPAStainR with hpar and our data, and found the issue was most likely due to the usage of simulated p-values in the chi-square analysis. To fix this, and overall improve the tool, we have changed the base test in HPAStainR() to a Fisher’s Exact Test, which works on the non-parametric data we have. From data not shown here, it does not lengthen the run time of HPAStainR(). Therefore all p-values in the paper have been updated to the results of the Fisher’s Exact Test.

  Output:

 We have updated the text to reflect the output of both `HPAStainR()` and `shiny_HPAStainR()` separately. If using the base `HPAStainR ()` function a tibble is returned, while the table returned in the `shiny_HPAStainR` is referred to as a Datatable.

 The p-values were character values due to the usage of `format.pvalue().` This has been revised and changed to numerical values for the next release.

 ##HPA data distribution

 We have also clarified Extended Table 1 and its relationship as the output to the ‘HPA_summary_maker()’ function.

 ## Confidence Score Calculation

 The creation of the “confidence scores” was strictly for our PanglaoDB analysis comparison and is not useful outside of validation studies. PanglaoDB had cell types with wildly variable numbers of marker genes. This caused cell types with 1 marker gene that had “high” staining (such as Schwann cells in extended table 4) to become a top hit in PanglaoDB (by staining score), but that does not reveal the accuracy of the tools as the marker genes may just be unique to the cell type in its specific tissue. Therefore we generated the confidence score, which controls and adjusts for the number of marker genes used because having ~40 proteins properly staining is more informative than ~2. The confidence scores are not used in the HPAStainR tool due to the higher consistency of protein staining per cell type, allowing the staining score to be sufficient for ranking.

 ## Cell type enriched p-value

 We have added more information explaining how the stringency is based on the “Reliability” column and how each level of stringency functions. The next release of HPAStainR will include this information in the description of the stringency parameter as well.

 ## The Panglao Database

 We have revised the table to include p-values of the tissue specificity. The reason we used confidence scores is that the p-values were simulated in the chi-square analysis used in HPAStainR, where there is a smaller range of possible p-values. We felt the confidence score was a better way to order the cell types due to its ability to highlight the staining score while controlling for how many genes each PanglaoDB cell type has.

 ## HPAStainR can help determine cell type populations in bulk RNA Sequencing

 In extended table 5 stomach glandular cells have the second-lowest adjusted p-value at 0.306 and in extended table 6 fallopian tube glandular cells, nasopharynx respiratory epithelial cells,  endometrium glandular cells, and cervix-uterine glandular cells all have an adjusted p-value <.05. However, the bronchus epithelial cell’s p-value is still the smallest at 1.58x10 ^-21^ compared to fallopian tube glandular cells at 3.99x10 ^-19^.

 ## Software availability

 All “old” versions have been removed and will remain so in the next version.

# Peer review: Reviewer response for version 1

**DOI:** 10.5256/f1000research.29559.r73228

**Published:** 2020-11-05

**Authors:** Mazdak Salavati

**Affiliations:** 1 The Roslin Institute and Royal (Dick) School of Veterinary Studies, University of Edinburgh, Edinburgh, UK

This manuscript describes a novel tool developed for extending access to the visual proteomics dataset produced by Human Protein Atlas (HPA). 

 The main two features that this shinyApp/R-package is as follows:

1. Enabling batch query of gene or protein name lists for cell type composition identification.
2. Use of bulk RNA data (tissues) in order to unravel cellular composition of the starting RNA sample. 

Authors have carried out an external validation with PanglaoDB human cell type dataset in order to confirm the soundness of both staining score and confidence score equations which are largely compatible with HPA cell type groups. They have also studied GTEx RNA-Seq (2 cell types) input gene lists with their pipeline which again was confirm by the top hit returned by the tools (albeit varying Staining score). 

 This tool has been developed very thoroughly and with a clear demand in the community at its design. However as highlighted by the authors in the conclusion section, one should approach subjective scored histochemistry obtained datasets always with caution. As the scoring bias introduced by scorers will remain as part of the rank outputs. 

 Suggestions for the authors: 

- I would highly recommend to include more data from GTEx tissues and expand the result section of your manuscript. 
- Perhaps consider a cross validation procedure in the future once more datasets are available for the same cell type. A 5-10 fold cross validation can immensely improve the reliability of the output ranked results. 
- Knowing a package like EnrichR that covers a variety of GSEA and Pathway enrichment databases, would it make sense to collaborate with their development team to expand functionality for cell type prediction through EnrichR? That's a question or challenge for the authors to answer or decide. 

Are the conclusions about the tool and its performance adequately supported by the findings presented in the article?

Yes

Is the rationale for developing the new software tool clearly explained?

Yes

Is the description of the software tool technically sound?

Yes

Are sufficient details of the code, methods and analysis (if applicable) provided to allow replication of the software development and its use by others?

Yes

Is sufficient information provided to allow interpretation of the expected output datasets and any results generated using the tool?

Yes

Reviewer Expertise:

Genetics and Genomics, Bioinfomatics, Cell biology.

I confirm that I have read this submission and believe that I have an appropriate level of expertise to confirm that it is of an acceptable scientific standard.

# Response

**Published:** 2021-03-12

**Authors:** Tim Nieuwenhuis

**Affiliations:** Johns Hopkins School of Medicine, USA

Thank you for reviewing our paper, below are our responses to your suggestions:

**Suggestion 1, Recommend to include more data from GTEx tissues**:

 This tool was generated to specifically analyze GTEx data. We are working on a project to use this tool for a more in-depth analysis of GTEx, similar to the lung paper cited.

**Suggestion 2, Cross-validation Procedure in the future once more datasets are available**:

 That is an excellent idea.  As more datasets become available, we will work to incorporate them into this tool.

**Suggestion 3, Collaborate with EnrichR**:

 Once HPAStainR package has fully matured through the review process, we will determine if it can be included in the excellent EnrichR tool.
